# Expression of MUC1 mucins inversely correlated with post-surgical survival of renal cell carcinoma patients

**DOI:** 10.1038/sj.bjc.6690355

**Published:** 1999-04

**Authors:** K Fujita, K Denda, M Yamamoto, T Matsumoto, M Fujime, T Irimura

**Affiliations:** Department of Urology, andFirst Department of Pathology, Juntendo University School of Medicine, Tokyo 113-8421, Japan; Laboratory of Cancer Biology and Molecular Immunology, Graduate School of Pharmaceutical Sciences, The University of Tokyo, 7-3-1 Hongo, Bunkyo-ku, Tokyo 113-0033, Japan

**Keywords:** renal cell carcinoma, metastasis, MUC1 mucin, prognosis

## Abstract

Surgical specimens of the normal kidney and of renal cell carcinoma (RCC) tissues at different stages of progression and of various histological grades were examined for the expression of MUC1 mucins with sialylated carbohydrates (sialylated MUC1 mucins) using a monoclonal antibody MY.1E12. Immunohistochemical studies revealed that the binding sites for this antibody were localized to the apical side of the epithelial cells of the distal convoluted tubules, Henle's loops and collecting ducts. However, proximal convoluted tubules, where RCC is considered to originate, were not stained. This antibody also bound strongly to RCC at advanced stages of progression and at metastatic sites, and to RCC of histologically high grades (undifferentiated). The epitope, presumably sialylated MUC1 mucin, was detected not only along the surface of the cell membranes but also in the cytoplasm. The level of expression of sialylated MUC1 mucins was inversely correlated with the survival of the patients with RCC and the disease-free survival period after curative surgery. Western blot analysis demonstrated that the electrophoretic mobility of sialylated MUC1 mucins of RCC was greater than that from the normal kidney. It is suggested that high levels of expression of sialylated MUC1 mucins in certain human RCC populations correlate with the aggressiveness of the disease, such as the tendency to form metastasis. © 1999 Cancer Research Campaign


*British Journal of Cancer* (1999) **80**(1/2), 301–308
© 1999 Cancer Research Campaign
Article no. bjoc.1998.0355

### Expression of MUC1 mucins inversely correlated with post-surgical survival of renal cell carcinoma patients

**K Fujita[1], K Denda[3], M Yamamoto[3], T Matsumoto[2], M Fujime[1] and T Irimura[3]**

[1]Department of Urology and [2]First Department of Pathology, Juntendo University School of Medicine, Tokyo 113-8421, Japan; [3]Laboratory of Cancer Biology and Molecular Immunology, Graduate School of Pharmaceutical Sciences, The University of Tokyo, 7-3-1 Hongo, Bunkyo-ku, Tokyo 113-0033, Japan

**Summary** Surgical specimens of the normal kidney and of renal cell carcinoma (RCC) tissues at different stages of progression and of various histological grades were examined for the expression of MUC1 mucins with sialylated carbohydrates (sialylated MUC1 mucins) using a monoclonal antibody MY.1E12. Immunohistochemical studies revealed that the binding sites for this antibody were localized to the apical side of the epithelial cells of the distal convoluted tubules, Henle's loops and collecting ducts. However, proximal convoluted tubules, where RCC is considered to originate, were not stained. This antibody also bound strongly to RCC at advanced stages of progression and at metastatic sites, and to RCC of histologically high grades (undifferentiated). The epitope, presumably sialylated MUC1 mucin, was detected not only along the surface of the cell membranes but also in the cytoplasm. The level of expression of sialylated MUC1 mucins was inversely correlated with the survival of the patients with RCC and the disease-free survival period after curative surgery. Western blot analysis demonstrated that the electrophoretic mobility of sialylated MUC1 mucins of RCC was greater than that from the normal kidney. It is suggested that high levels of expression of sialylated MUC1 mucins in certain human RCC populations correlate with the aggressiveness of the disease, such as the tendency to form metastasis.

**Keywords:** renal cell carcinoma; metastasis; MUC1 mucin; prognosis

Approximately 30% of patients with renal cell carcinoma (RCC) have evidence of metastasis at the time of diagnosis. In a significant number of patients, the metastasis occurs after surgery with curative intent. The most common target organs of metastasis are the lung, bone, liver and lymph nodes (Holland, 1973). Chemotherapy and radiotherapy are not effective in patients with metastatic RCC (van der Werf-Messing, 1973; Fojo et al, 1987). Administration of biologic response modifiers such as interferon (IFN)-α, IFN-γ and interleukin-2 elicited responses in 10–27% of the patients, but the duration of the response was limited (Umeda and Niijima, 1986; Sarna et al, 1987; Dreicer and Williams, 1995). Consequently, the presence or emergence of metastasis indicates a dismal prognosis, and the 5-year survival rate of RCC patients with metastasis is reported to be less than 20% (Dreicer and Williams, 1995; Giberti et al, 1997).

It is thought that not all tumour cells in a primary lesion are able to metastasize to distant organs. Tumour cells with a high metastatic potential arise within primary tumours at an early stage and become predominant during the progression of the disease to an advanced stage (Poste and Fidler, 1980; Irimura et al, 1993). The glycoproteins expressed on the tumour cells of a metastatic phenotype are qualitatively and quantitatively different from those of the non-metastatic phenotype (Nicolson, 1982; Irimura and Reading, 1987; Matsushita et al, 1990; Irimura et al, 1991). Some altered glycoproteins presumably affect the behaviour of the cell during the process of metastasis, i.e. release of tumour cells from the primary lesion, invasion of vessels, adhesion to endothelial cells and escape from immune recognition (Ponta et al, 1994). Detection of such altered glycoproteins in surgical specimens may provide information regarding the potential of metastasis after the surgery.

MUC1 mucin (also known as episialin, PUM, PEM and epitectin) is one of the glycoproteins expressed by the metastatic phenotype (Hilkens et al, 1992; Nakamori et al, 1994; Gendler and Spicer, 1995). MUC1 mucin is a transmembrane glycoprotein with a large extracellular domain that extends 200–500 nm above the cell membrane. The protein backbone of the extracellular domain consists of 30–90 repeats of 20 amino acids that are nearly identical (Gendler et al, 1990; Lan et al, 1990; Hilkens et al, 1992). Proline residues make the protein backbone straight, and serine and threonine residues, which are *O*-glycosylated, make the structure very rigid (Jentoft, 1990). These *O*-linked glycans are fully sialylated during the maturation of MUC1 mucin through a *trans*-Golgi network (Litvinov and Hilkens, 1993).

Sialylated MUC1 mucin expressed on cancer cells suppresses homotypic cellular aggregation (Ligtenberg et al, 1992) and cell-matrix adhesion and promotes invasion in Matrigel (Hilkens et al, 1993). Moreover, sialylated MUC1 mucin inhibits cytotoxic lymphocyte–target cell interactions in vitro (Irimura et al, 1990). These molecules also induced apoptosis of lymphocytes (Gimmi et al, 1996; Agrawal et al, 1998). Thus, the findings to date suggest that cancer cells in vivo with a high level of expression of sialylated MUC1 mucin may be able to detach easily from the primary lesion and survive in circulation or in distant organs of metastasis by escaping from immune surveillance.

*Received 20 April 1998*
*Revised 2 September 1998*
*Accepted 11 September 1998*

*Correspondence to:* T Irimura

Previously, we generated a novel monoclonal antibody (mAb), MY.1E12, which preferentially recognizes sialylated MUC1 mucin (Yamamoto et al, 1996). In this study, we used mAb MY.1E12 to detect sialylated MUC1 mucin in formalin-fixed, paraffin-embedded surgical specimens from RCC of various histological grades and at different stages of progression. A correlation between the level of expression of sialylated MUC1 mucin and the survival of patients was demonstrated.

### MATERIALS AND METHODS

### Specimens

Fifty-one specimens of RCC were obtained from patients who had undergone radical nephrectomy at Juntendo University. All specimens were classified by pathologists according to the TNM system (International Union Against Cancer, 1987) UICC staging (International Union Against Cancer, 1992) and pathological grading based on the nuclear atypia (Japanese Urological Association, 1992). In half of the cases, the renal cortex, medulla and carcinoma were sampled before formalin fixation and were kept frozen at –80°C until use. Specimens from metastatic sites were also obtained from autopsy cases.

### A cell line and a mAb

Capan-1 pancreatic adenocarcinoma cell lines were obtained from Dr Marsha L Frazier of the Department of Epidemiology, The University of Texas, MD Anderson Cancer Center, Houston, TX, USA. The cells were maintained in a 1:1 mixture of Dulbecco's modified Eagle's medium and Ham's F12 medium that contained 10% fetal calf serum in a humidified atmosphere that contained 5% carbon dioxide at 37°C. mAb MY.1E12 (IgG2a), specific for MUC1 mucin with sialylated *O*-linked oligosaccharides, was prepared as described previously (Yamamoto et al, 1996). The epitope structure recognized by this mAb was previously described (Yamamoto et al, 1996).

### Immunohistochemical staining

Surgical specimens fixed in formalin and embedded in paraffin were cut at 4 μm. The sections were deparaffinized and treated with 0.3% hydrogen peroxide in methanol for 30 min to block endogenous peroxidase activity. The sections were rehydrated in phosphate-buffered saline (PBS) and incubated with 2% bovine serum albumin (BSA) in PBS for 40 min at room temperature (r.t.). mAb MY.1E12 (culture supernatant of the hybridoma cells) was diluted 1:1 in PBS containing 2% BSA. The sections were incubated with the diluted mAb MY.1E12 for 1 h at 37°C. After washing with PBS, the sections were incubated with biotinylated goat anti-mouse immunoglobulin (DAKO JAPAN Co., Ltd, Kyoto, Japan) for 40 min at r.t. The specimens were washed with PBS and incubated with a solution of horseradish peroxidase-conjugated streptavidin (DAKO JAPAN Co., Ltd) for 40 min at r.t. After repeated washing, they were treated with DAB reagent, which consisted of a 0.05% solution of 3,3′-diaminobenzidine (Sigma, St Louis, MO, USA) dissolved in 10 ml of PBS and 3 μl of 30% hydrogen peroxide. The specimens were counterstained with haematoxylin.

In some experiments, deparaffinized and hydrogen peroxide-treated sections were incubated with sialidase (*Vibrio cholerae*, 50 mU ml$^{-1}$ in PBS; Calbiochem, La Jolla, CA, USA) at 37°C for 3 h (Matsushita et al, 1990). After several washings with PBS, the sections were treated with mAb MY.1E12 and processed as described above.

### Western blot analysis

The frozen surgical specimens were thawed and homogenized with 0.5% Nonidet P-40 in a desalting buffer that consisted of 250 mM sucrose, 10 mM Tris-HCl, 50 μM calcium chloride and 10 mM phenylmethylsulphonyl fluoride, pH 7.2. The Capan-1 cells were also lysed in the same buffer. The supernatants of the lysates were mixed with one half volume of 187.5 mM Tris-HCl that contained 5% sodium dodecyl sulphate, 3% 2-mercaptoethanol, 30% glycerol and 1.5 mM EDTA. The mixtures containing 200 μg of protein were heated for 5 min at 100°C and then electrophoretically separated on 4% polyacrylamide gels in the presence of 0.1% sodium dodecyl sulphate (SDS-PAGE). They were transblotted onto polyvinylidene difluoride membranes (Immobilon; Millipore, Boston, MA, USA). The membranes were incubated in 2% BSA in PBS at 4°C overnight and then mixed with mAb MY.1E12 (culture supernatant of hybridoma cells) diluted in an equal volume of 2% BSA for 2 h at r.t. After washing with PBS that contained 0.1% Tween-20, they were incubated with rabbit anti-mouse IgG labelled with alkaline phosphatase (Zymed Laboratories Inc., San Francisco, CA, USA) for 1 h at r.t. The membranes were washed with PBS that contained 0.1% Tween-20 and then treated with ECL (Amersham, Buckinghamshire, UK) to visualize the antibodies that were bound.

### Statistical analysis

Statistical analysis was performed using Student's *t*-test. The survival of patients and recurrence of disease were recorded every month. Patient survival was analysed by the method of Kaplan and Meier (1977). Disease-free survival of patients undergoing curative surgery was analysed by the same method. Differences in the survival of patients in subgroups that were classified according to reactivity with mAb MY.1E12 were analysed by the log-rank test.

### RESULTS

### Immunohistochemical localization of sialylated MUC1 mucin in normal kidney

The luminal surfaces of the distal convoluted tubules, collecting ducts and Henle's loops were strongly stained with mAb MY.1E12, but the proximal convoluted tubules and Bowman's capsules were not (Figure 1). This reactivity with mAb MY.1E12 was completely abolished by sialidase treatment of the specimens (data not shown).

### Immunohistochemical localization of sialylated MUC1 mucin in RCC

The staining pattern of mAb MY.1E12 in RCC demonstrated that the distribution of sialylated MUC1 mucin in the tumour was heterogeneous. A cell was judged to be positively stained when either the cytoplasm or the whole cell membrane was stained. Conversely, when only the apical side of the membrane was stained it was judged to be negative. To quantitate the degree of reactivity with mAb MY.1E12, the percentage of positively

 © Cancer Research Campaign 1999

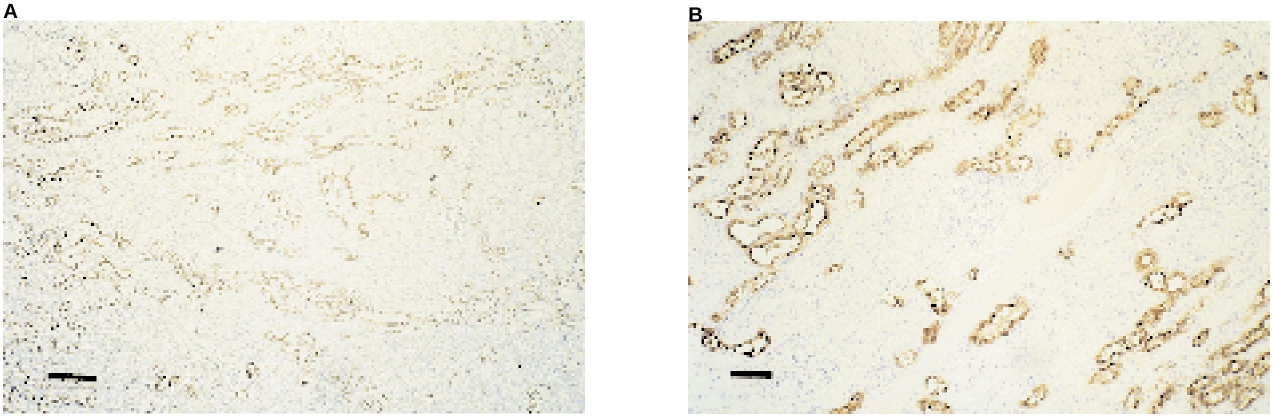

**Figure 1** Immunohistochemical staining of normal kidney tissues with mAb MY.1E12. Sialylated MUC1 mucins were detected in Henle's loops, distal convoluted tubules and collecting ducts. Bars at the bottom left of the panels indicate 300 µm for (**A**) and 100 µm for (**B**), respectively

**Table 1** Expression of sialylated MUCI mucin in 51 RCC specimens

| Variables | No. | Positive rate[a] (mean ± s.d.) | Difference | Probability |
|---|---|---|---|---|
| Age | | | | |
| < 60 | 28 | 56.6 ± 45.5 | | |
| | | | ≥ 60 vs < 60 | NS[b] |
| ≥ 60 | 23 | 54.7 ± 43.2 | | |
| Sex | | | | |
| Male | 37 | 52.5 ± 44.4 | | |
| | | | Male vs Female | NS |
| Female | 14 | 64.2 ± 43.4 | | |
| Size of tumour | | | | |
| < 6.0 cm | 21 | 42.3 ± 45.0 | | |
| | | | ≥ 6.0 cm vs < 6.0 cm | NS |
| ≥ 6.0 cm | 30 | 65.1 ± 41.5 | | |
| T classification | | | | |
| T1 | 2 | 10.0 ± 14.1 | | |
| T2 | 30 | 52.6 ± 45.4 | | |
| | | | T1 + T2 vs T3 + T4 | NS |
| T3 | 17 | 61.4 ± 41.8 | | |
| T4 | 2 | 100 ± 0 | | |
| N classification | | | | |
| N0 | 42 | 50.1 ± 44.4 | | |
| | | | N0 vs N1–3 | $P < 0.05$ |
| N1–3 | 9 | 82.2 ± 32.7 | | |
| M classification | | | | |
| M0 | 32 | 37.3 ± 43.8 | | |
| | | | M0 vs M1 | $P < 0.001$ |
| M1 | 19 | 86.8 ± 21.6 | | |
| Pathological grading | | | | |
| Grade 1 | 21 | 22.8 ± 36.7 | | |
| | | | Grade 1 vs Grade 2 + Grade 3 | $P < 0.001$ |
| Grade 2 | 17 | 75.8 ± 32.4 | | |
| | | | Grade 1 + Grade 2 vs Grade 3 | $P < 0.05$ |
| Grade 3 | 13 | 82.6 ± 34.4 | | |
| UICC staging | | | | |
| Stage 1 | 2 | 10.0 ± 14.1 | | |
| | | | Stage 1 vs Stage 2 + 3 + 4 | NS |
| Stage 2 | 19 | 31.5 ± 42.1 | | |
| | | | Stage 1 + 2 vs Stage 3 + 4 | $P < 0.001$ |
| Stage 3 | 11 | 52.2 ± 47.6 | | |
| | | | Stage 1 + 2 + 3 vs Stage 4 | $P < 0.001$ |
| Stage 4 | 19 | 86.8 ± 21.6 | | |

[a]The percentage of positively stained cells in the tumour cells. [b]Not significant.

© Cancer Research Campaign 1999 

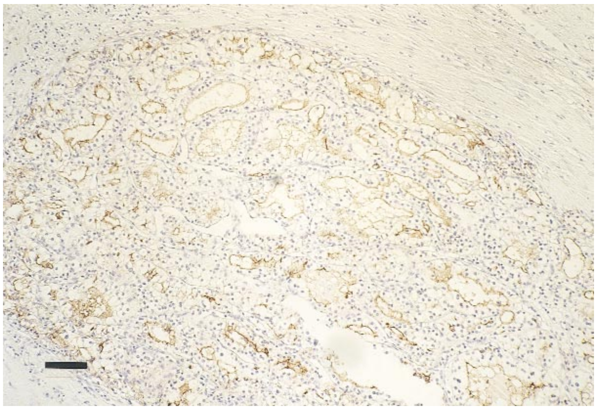

**Figure 2** Immunohistochemical staining of grade 1 RCC with mAb MY.1E12. A bar at the bottom left of the panel corresponds to 100 µm

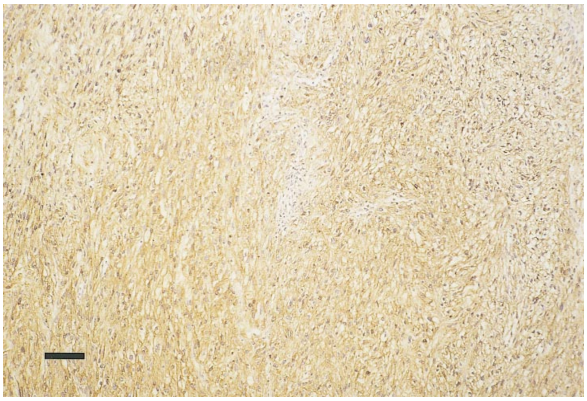

**Figure 4** Immunohistochemical staining of grade 3 RCC with mAb MY.1E12. A bar at the bottom left of the panel corresponds to 100 µm

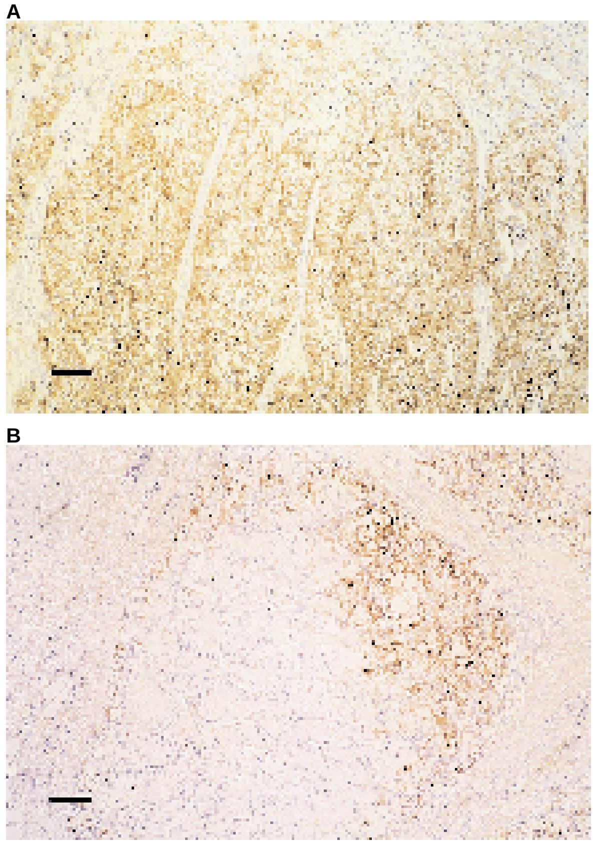

**Figure 3** Immunohistochemical staining of grade 2 RCC with mAb MY.1E12. Bars at the bottom left of the panels correspond to 100 µm for (**A**) and (**B**)

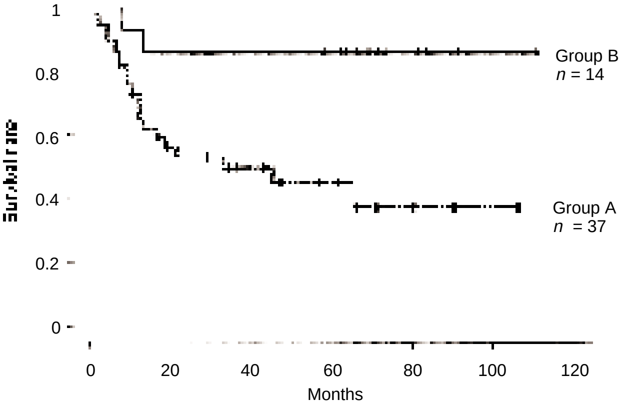

**Figure 5** Relationship between the prognosis and expression of sialylated MUC1 mucins in the RCC specimens by Kaplan–Meier's survival curve. The patients with RCC were divided into two groups according to the percentage of stained cells in the cancer specimens: Group A, which had greater or equal to 5% of the cells stained, and Group B, which had less than 5% of the cells stained ($P$ = 0.009 according to the log-rank test)

stained cells in all tumour cells (positive rate) was determined for each specimen. The levels of expression of sialylated MUC1 mucin in 51 surgical specimens of RCC are summarized in Table 1.

Primary tumours that had already metastasized to distant organs (M1) were stained more strongly than tumours that had not metastasized (M0) ($P$ < 0.001). Moreover, primary tumours that had metastasized to the regional lymph nodes (pN1–3) were also stained more strongly than the tumours that had not (pN0) ($P$ < 0.05). No difference in reactivity was found between tumours that were limited to the kidney (pT1–2) and those that had invaded beyond the kidney (pT3–4). Reactivity to the antibody was not affected by the size of the tumour or the age and sex of the patients. RCC at UICC stage 3 or 4 (advanced stage of cancer progression) showed higher expression of sialylated MUC1 mucin than RCC at stage 1 or 2 ($P$ < 0.001).

This pathologic grading used in the study presented here is based on the nuclear atypia of the tumour. Grade 1 tumours, which had nuclei resembling the nuclei of proximal convoluted tubule cells, showed weak reactivity with the mAb (Figure 2). Grade 2 tumours, which had larger or irregular nuclei with nucleoli, showed moderate reactivity (Figure 3). Grade 3 tumours, which had bizarre or giant nuclei, reacted strongly with the mAb (Figure 4). Sialidase treatment completely abolished the reactivity of the RCC specimens with mAb MY.1E12.

Tissues of metastasis of distant organs were obtained from six autopsy cases. The immunohistochemical reactivity to MY.1E12 was high for all the tissues (positive rate: 69.0 ± 40.4).

 © Cancer Research Campaign 1999

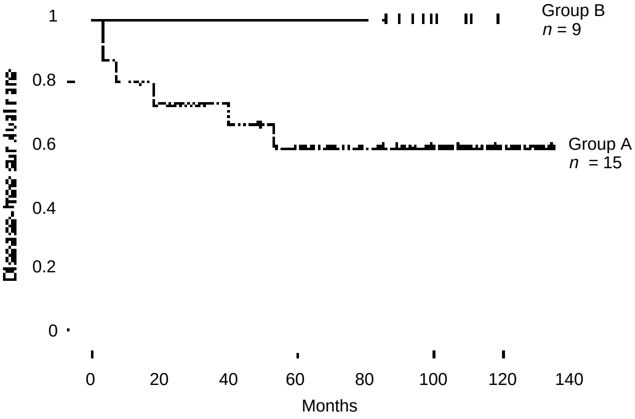

**Figure 6** Relationship between disease-free survival and expression of sialylated MUC1 mucins in the RCC specimens by Kaplan–Meier's survival curve. Groups A and B represent the same categories as in Figure 5 ($P$ = 0.034 according to the log-rank test)

### Relationship between the prognosis or disease-free survival of patients with RCC and expression of sialylated MUC1 mucin in the specimens

The patients with RCC were divided into two groups according to the percentage of stained cells in the cancer specimens: Group A, which had greater or equal to 5% of the cells stained, and Group B, which had less than 5% of the cells stained. The survival curves of the patients in these two groups were drawn by the method of Kaplan–Meier (Figure 5). The prognosis of the patients in Group A was significantly poorer than that of the patients in Group B ($P$ = 0.009). The 5-year survival rate was 47.2% in Group A and 85.7% in Group B. The disease-free survival of patients who had undergone curative surgery is shown in Figure 6. It should be noted that none of the patients subjected to this comparison had detectable metastasis at the time of surgery. The patients in Group A developed metastasis more frequently than the patients in Group B ($P$ = 0.034). The survival curves were also compared within restricted categories. In Figure 7A, a total of 27 patients who did not have detectable metastasis at the time of surgery (N0M0) were compared according to mAb MY.1E12 binding. The difference between the MUC1 mucin-positive group and MUC1 mucin-negative group was statistically significant ($P$ = 0.043). When a total of 32 patients categorized as T1 and T2 (confined tumours) were compared, significant difference in the survival between patients with stained tumours and unstained tumours was also observed ($P$ = 0.010) (Figure 7B). Patients with MUC1 mucin-positive tumours showed significantly poorer survival than those with MUC1 mucin-negative tumours ($P$ = 0.014), when patients with tumour cells having grade 1 and grade 2 nuclear atypia were examined (Figure 7C).

### Western blot analysis of sialylated MUC1 mucin in normal kidney, RCC and Capan-1 cell lines

Normal kidney tissues were obtained from an area approximately 3 cm from the primary RCC. The cortex and medulla of the normal tissue were separated macroscopically. The cortex contained distal and proximal convoluted tubules and Bowman's capsules. The medulla contained collecting ducts and Henle's loops. Sialylated

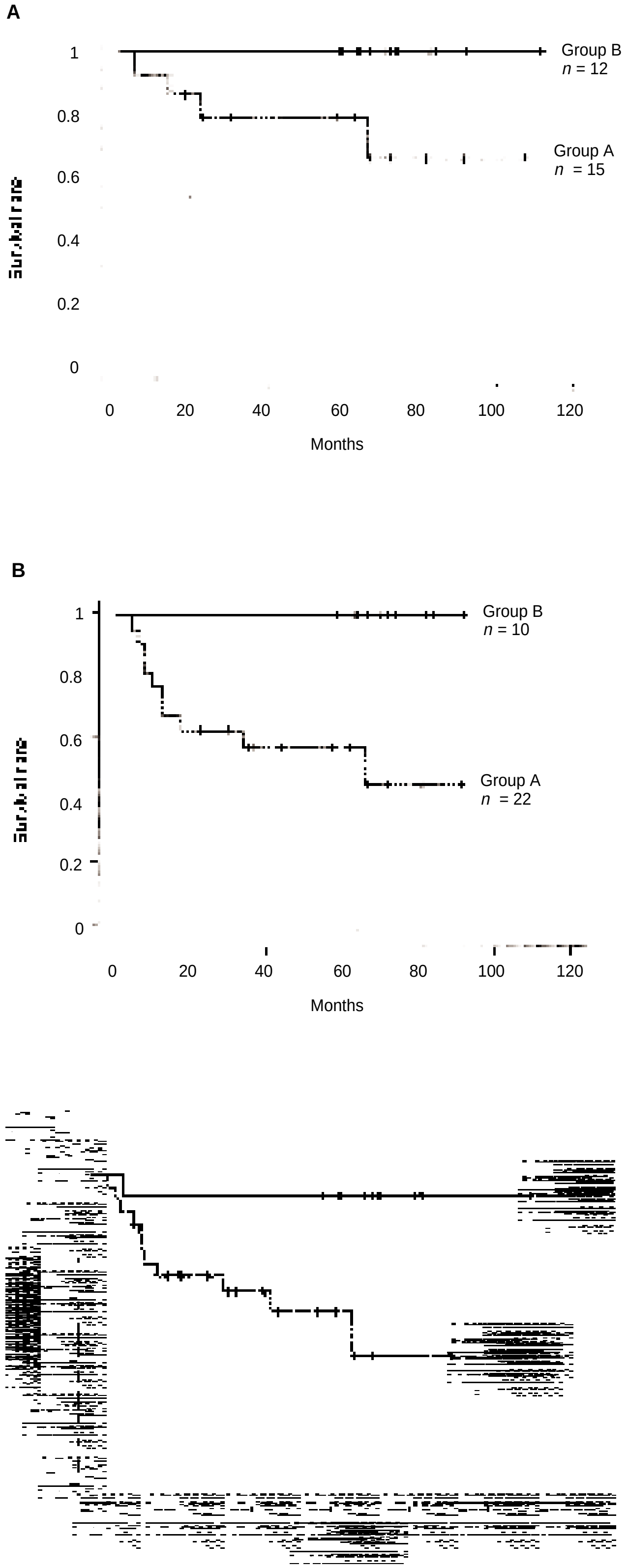

**Figure 7** Relationship between prognosis and mAb MY.1E12 binding to RCC tissues by Kaplan–Meier's survival curve within three subcategories of patients. (**A**) Patients classified as N0M0 ($P$ = 0.043 according to the log-rank test). (**B**) Patients classified as T1 and T2 ($P$ = 0.010 according to the log-rank test). (**C**) Patients classified as grade 1 and grade 2 ($P$ = 0.014 according to the log-rank test). Groups A and B represent the same categories as in Figure 5

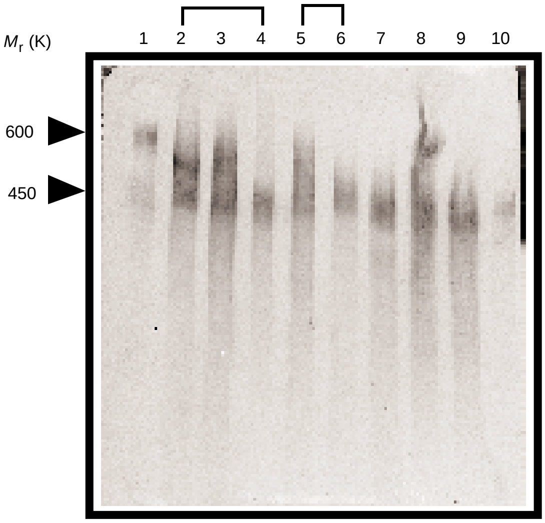

**Figure 8** Binding of mAb MY.1E12 to electrophoretically separated lysates of normal kidney tissues, RCC and Capan-1 cells. Lane 1 is the lysate of Capan-1 cells. Lane 2 is the lysate of the normal medulla of the kidney, which contains Henle's loops and collecting ducts. Lane 3 is the lysate of the normal cortex of the kidney, which contains Bowman's capsules, proximal convoluted tubules and distal convoluted tubules. Lane 4 is the lysate of grade 2, pT3N0M1 RCC. Lane 5 is the lysate of the normal cortex of the kidney. Lane 6 is the lysate of grade 2, pT3N0M0 RCC. Lane 7 is the lysate of grade 1, pT2N0M0 RCC. Lane 8 is the lysate of grade 3, pT3N1M1 RCC. Lane 9 is the lysate of grade 2, pT2N0M1 RCC. Lane 10 is the lysate of grade 3, pT2N0M1 RCC. These cell lysates were subjected to SDS-PAGE using 4% gels, followed by blotting onto polyvinylidene difluoride membranes. The membranes were processed for detection of sialylated MUC1 mucin, as described in Materials and Methods. Samples obtained from the same patient are bracketed

MUC1 mucin in the lysates of the normal cortex and medulla, RCC and Capan-1 cell lines was detected with mAb MY.1E12 after SDS-PAGE (Figure 8). When normal tissue and RCC from the same patients were compared, normal tissue contained components with slower mobility than RCC. RCC and Capan-1 cells from one patient (lane 8) apparently contained two distinct bands corresponding to two alleles of MUC1 gene. Sialylated MUC1 mucin of RCC from five patients (corresponding to lanes 4, 6, 7, 9 and 10) had similar electrophoretic migration corresponding to an apparent $M_r$ 400 000.

### DISCUSSION

The expression of MUC1 mucin by RCC at various stages of progression was demonstrated. Primary lesions with distant organ metastasis (M1) or lymph node metastasis (pN1–3) were stained more strongly by the mAb specific for sialylated MUC1 mucin than lesions without metastasis ($P < 0.001$ and 0.05 respectively). Among patients who had undergone surgery with curative intent for localized cancer (pT1–3, pN0, M0), the patients with a high level of MUC1 mucin expression had a poorer disease-free survival rate than the patients with a low level of MUC1 mucin expression ($P < 0.05$). These results indicate that RCC cells with high MUC1 mucin expression have a strong potential for metastasis, and that micrometastases may already exist at the time of surgery if the expression of MUC1 mucin was high at the primary lesion. There were no significant differences in MUC1 mucin expression between cancer that was confined within the kidney (pT1–2) and cancer that was invasive through the renal capsule into the perinephric fat (pT3–4). The size of the tumour did not affect the expression of MUC1 mucin. These results are consistent with our previous findings on colon carcinoma cells indicating that primary tumours at advanced stages expressed increased levels of MUC1 mucin (Nakamori et al, 1994).

RCC cells were histologically divided into three grades based on the degree of nuclear atypia (Japanese Urological Association, 1992). RCCs in grade 1 (well-differentiated) had nuclei which resembled that of proximal convoluted tubules. These RCCs expressed MUC1 mucin weakly and the cells sometimes preserved the polar nature of its localization. Most RCCs in grade 2 or 3 (moderately or poorly differentiated) expressed MUC1 mucin strongly at the surface of the cell membranes and in the cytoplasm. Due to such strong association with histological grades and stages of the disease, the increased sialylated MUC1 mucin revealed by mAb MY.1E12 is not considered to be an independent prognostic marker. However, this phenotype should serve as a unique biological feature associated with advanced RCC.

MUC1 mucin is a long and rigid glycoprotein that extrudes out of the cell membrane and is negatively charged due to the addition of sialic acids. In normal glandular cells, MUC1 mucin is expressed only on the apical (luminal) side of the cells. The charge repulsion caused by the sialic acids and the rigid structures of the mucins apparently maintain the lumen of the gland and prevent attachment of foreign molecules or cells. In most cancer cells, this polarization is lost and overexpression of MUC1 mucin on the whole cell membrane is believed to destabilize the cell–cell adhesion and to permit cancer cells to migrate and metastasize (Hilkens et al, 1992). This tendency to lose homotypic cell adhesion was experimentally observed under the influence of a potent adhesion molecule, E-cadherin, as reported by Hilkens and co-workers. They used double transfectant cells that expressed both MUC1 mucin and E-cadherin to show that MUC1 mucin could prevent intercellular adhesion mediated by E-cadherin (Hilkens et al, 1993; Wesseling et al, 1996). In these studies, however, sialic acid at the termini of carbohydrate chains did not seem to play roles. Although the present study utilized with a mAb specific for sialylated MUC1 mucin, our preliminary experiments with mAb specific for core polypeptide portions of MUC1 mucin also showed similar differences in their bindings to low- and high-grade RCC. A decrease in E-cadherin function was proposed to favour the detachment of cells in most carcinomas, resulting in a more aggressive tumour (Takeichi, 1991; Birchmeier and Behrens, 1994). However, the frequency of expression of E-cadherin in RCC seems to be very low regardless of the grade. Even low-grade RCCs do not express E-cadherin. Furthermore, the normal proximal convoluted tubules, where RCC is considered to originate (Oosterwijk et al, 1986; Mackay et al, 1987), do not express E-cadherin (Terpe et al, 1993; Katagiri et al, 1995). Therefore, MUC1 mucin may be antagonizing other adhesion molecules in RCC.

As stated above, RCC originates from the proximal convoluted tubules. Our immunohistochemical study of normal kidney tissues demonstrated that MUC1 mucin is absent in the proximal convoluted tubules, but present on cell surfaces of the distal convoluted tubules, collecting ducts and Henle's loops. MUC1 mucin is detected mainly on the apical side of these cells. In the process of malignant transformation from the proximal convoluted tubules, RCC cells acquire the ability to express MUC1 mucin.

 © Cancer Research Campaign 1999

Electrophoretic analysis revealed slight differences in the apparent $M_r$ of MUC1 mucin expressed in malignant cells and normal cells (collecting ducts, distal convoluted tubules and Henle's loops). Such altered glycosylation and sialylation in RCC might influence the reactivity of MUC1 mucin with MY.1E12. Thus, there is a possibility that this antibody does not react with a portion of a mixture of diversely glycosylated MUC1 mucin, resulting in unique banding patterns revealed by Western blot analysis. It remains to be elucidated whether these differential migrations were due to changes in the number of *O*-linked carbohydrate chains or extension of carbohydrate chains.

van der Wiel-van Kemenade et al (1993) reported that melanoma cells transfected with MUC1 genes are resistant to killing by cytotoxic T-cells and lymphokine-activated killer cells. We reported previously that colon carcinoma cells with high MUC1 mucin production are less sensitive to cytolysis by lymphokine-activated killer cells (Irimura et al, 1990). These data suggest that a greater amount of MUC1 mucin on RCC cells may conceal putative recognition sites for these killer lymphocytes. This would increase the chance of survival and colonization of RCC cells by allowing them to evade immune surveillance. It is also possible that the alteration in MUC1 mucin expression is simply associated with the status of differentiation of RCC and may not influence the cells' malignant behavior.

The mechanistic basis for the increase of mAb MY.1E12-reactive MUC1 mucin in high-grade RCC is not known. Host micro-environmental factors may affect the production of MUC1 mucin. We reported that a soluble factor, produced by connective tissue of the colon, increased the expression of MUC1 mucin by colon carcinoma cells in vitro (Irimura et al, 1990; Dohi et al, 1993; Shirotani et al, 1994). A higher level of expression of MUC1 mucin was observed in RCC cells adjacent to the connective tissue of the kidney (Figure 3B), suggesting that a similar protein that increases expression of MUC1 mucin may be produced by the interstitium of the kidney.

### ACKNOWLEDGEMENTS

We thank Dr Shinji Nakamura of Juntendo University School of Medicine for his assistance on the immunohistochemical staining, Ms Susan Custead of the University of Texas MD Anderson Cancer Center for her editorial reading of this manuscript, and Ms Chizu Hiraiwa for her assistance in preparing this manuscript. This work was supported by grants-in-aid from the Ministry of Education, Science, Sports and Culture of Japan (05274101, 05557104, 07407063, and 0771322), the Ministry of Health and Welfare, the Japan Health Science Foundation, the Research Association for Biotechnology, PROBRAIN and the New Energy and Industrial Technology Development Organization.

### REFERENCES

Agrawal B, Klantz MJ, Reddish MA and Longenecker BM (1998) Cancer-associated MUC1 mucin inhibits human T-cell proliferation, which is reversible by IL-2. *Nature Med* **4**: 43–49

Birchmeier W and Behrens J (1994) Cadherin expression in carcinomas: role in the formation of cell junctions and the prevention of invasiveness. *Biochim Biophys Acta* **1198**: 11–26

Dohi DF, Sutton RC, Frazier ML, Nakamori S, McIsaac AM and Irimura T (1993) Regulation of sialomucin production in colon carcinoma cells. *J Biol Chem* **268**: 10133–10138

Dreicer R and Williams RD (1995) Renal parenchymal neoplasms. In *Smith's General Urology*, Tanagho EA and McAninch JW (eds), pp. 372–391. Appleton & Lange: East Norwalk

Fojo AT, Ueda K, Slamon DJ, Poplack DG, Gottesman MM and Pastan I (1987) Expression of multidrug-resistance gene in human tumors and tissues. *Proc Natl Acad Sci USA* **84**: 265–269

Gendler SJ, Lancaster CA, Taylor-Papadimitriou J, Duhig T, Peat N, Burchell J, Pemberton L, Lalani E and Wilson D (1990) Molecular cloning and expression of human tumor-associated polymorphic epithelial mucin. *J Biol Chem* **265**: 15286–15293

Gendler SJ and Spicer AP (1995) Epithelial mucin genes. *Ann Rev Physiol* **57**: 607–634

Giberti C, Oneto F, Martorana G, Rovida S and Carmignani G (1997) Radical nephrectomy for renal cell carcinoma: long-term results and prognostic factors on a series of 328 cases. *Eur Urol* **31**: 40–48

Gimmi CD, Morrison BW, Mainprice BA, Gribben JG, Boussiotis VA, Freeman GJ, Park SYL, Watanabe M, Gong J, Hayes DF, Kufe DW and Nadler LM (1996) Breast cancer-associated antigen, DF3/MUC1, induces apoptosis of activated human T cells. *Nature Med* **2**: 1367–1370

Hilkens J, Ligtenberg MJ, Vos HL and Litvinov SV (1992) Cell membrane-associated mucins and their adhesion-modulating property. *Trends Biochem Sci* **17**: 359–363

Hilkens J, Wesseling J, Vos HL, Litvinov SL, Boer M, van der Valk S, Calafat J, van der Wiel-van Kemenade E and Figdor C (1993) Episialin modulates cell–cell and cell–matrix adhesion, promotes invasion in matrigel and inhibits cytolysis by cytotoxic effector cells. In *Biology of Vitronectins and their Receptors*, Preissner KT, Rosenblatt S, Kost C, Wegerhoff J and Mosher DF (eds), pp. 193–200. Excerpta Medica: Amsterdam

Holland JM (1973) Cancer of the kidney. Natural history and staging. *Cancer* **32**: 1030–1042

International Union Against Cancer (1987) *TNM Classification of Malignant Tumors*, 4th Edn, Hermanek P and Sobin LH (eds). Springer-Verlag: Berlin

International Union Against Cancer (1992) TNM Atlas, 3rd Edn, 2nd Revision, Spiessl B, Beahrs OH, Hermanek P, Hutter RVP, Scheibe O, Sobin LH and Wagner G (eds). Springer-Verlag: Berlin

Irimura T and Reading CL (1987) Surface properties of metastatic tumor cells. *Cancer Bull* **39**: 132–141

Irimura T, McIsaac AM, Carlson DA, Yagita M, Grimm EA, Menter DG, Ota DM and Cleary KR (1990) Soluble factor in normal tissues that stimulates high-molecular-weight sialoglycoprotein production by human colon carcinoma cells. *Cancer Res* **50**: 3331–3338

Irimura T, Matsushita Y, Hoff SD, Yamori T, Nakamori S, Frazier ML, Giacco GG, Clearly KR and Ota DM (1991) Ectopic expression of mucins in colorectal cancer metastasis. *Semin Cancer Biol* **2**: 129–139

Irimura T, Nakamori S, Matsushita Y, Taniuchi Y, Todoroki N, Tsuji T, Izumi Y, Kawamura Y, Hoff SD, Cleary KR and Ota DM (1993) Colorectal cancer metastasis determined by carbohydrate-mediated cell adhesion: role of sialyl-$Le^x$ antigens. *Semin Cancer Biol* **4**: 319–324

Japanese Urological Association, The Japanese Society of Pathology and Japan Radiological Society (1992) *General Rules for Clinical and Pathological Studies on Renal Cell Carcinoma*, 2nd Edn, pp. 2–105. Kanehara Shuppan: Tokyo

Jentoft N (1990) Why are proteins O-glycosylated? *Trends Biochem Sci* **15**: 291–294

Kaplan EL and Meier P (1977) Non-parametric estimation from incomplete observation. *J Am Stat Assoc* **16**: 95–101

Katagiri A, Watanabe R and Tomita Y (1995) E-cadherin expression in renal cell cancer and its significance in metastasis and survival. *Br J Cancer* **71**: 376–379

Lan MS, Barta SK, Qi W-N, Metzgar RS and Hollingsworth MA (1990) Cloning and sequencing of a human pancreatic tumor mucin cDNA. *J Biol Chem* **265**: 15294–15299

Ligtenberg MJ, Buijs F, Vos HL and Hilkens J (1992) Suppression of cellular aggregation by high levels of episialin. *Cancer Res* **52**: 2318–2234

Litvinov SV and Hilkens J (1993) The epithelial sialomucin, episialin, is sialylated during recycling. *J Biol Chem* **268**: 21364–21371

Mackay B, Ordonez NG, Khoursand J and Bennington JL (1987) The ultrastructure and immunocytochemistry of renal cell carcinoma. *Ultrastruct Pathol* **11**: 483–502

Matsushita Y, Cleary KR, Ota DM, Hoff SD and Irimura T (1990) Sialyl-dimeric Lewis-X antigen expressed on mucin-like glycoproteins in colorectal cancer metastases. *Lab Invest* **63**: 780–791

Nakamori S, Ota DM, Cleary KR, Shirotani K and Irimura T (1994) MUC1 mucin expression as a marker of progression and metastasis of human colorectal carcinoma. *Gastroenterology* **106**: 353–361

© Cancer Research Campaign 1999

Nicolson GL (1982) Cancer metastasis. Organ colonization and cell surface properties of malignant cells. *Biochim Biophys Acta* **695**: 113–176

Oosterwijk E, Ruiter DJ, Wakka JC, Huiskens-van der Meij JW, Jonas U, Fleuren GJ, Zwartendijk J, Hoedemaeker P and Warnaar SO (1986) Immunohistochemical analysis of monoclonal antibodies to renal antigens. Application in the diagnosis of renal cell carcinoma. *Am J Pathol* **123**: 301–309

Ponta H, Sleeman J and Herrlich P (1994) Tumor metastasis formation: cell-surface proteins confer metastasis-promoting or -suppressing properties. *Biochim Biophys Acta* **1198**: 1–10

Poste G and Fidler IJ (1980) The pathogenesis of cancer metastasis. *Nature* **283**: 139–146

Sarna G, Figlin R and deKernion J (1987) Interferon in renal cell carcinoma. The UCLA experience. *Cancer* **59**: 610–612

Shirotani K, Taylor-Papadimitriou J, Gendler SJ and Irimura T (1994) Transcriptional regulation of the MUC1 mucin gene in colon carcinoma cells by a soluble factor. Identification of a regulatory element. *J Biol Chem* **269**: 15030–15035

Takeichi M (1991) Cadherin cell adhesion receptors as a morphogenetic regulator. *Science* **251**: 1451–1455

Terpe H-J, Tajrobehkar K, Günthert U and Altmannsberger M (1993) Expression of cell adhesion molecules alpha-2, alpha-5 and alpha-6 integrin, E-cadherin, N-CAM and CD-44 in renal cell carcinomas. *Virchows Arch* **422**: 219–224

Umeda T and Niijima T (1986) Phase II study of alpha interferon on renal cell carcinoma. Summary of three collaborative trials. *Cancer* **58**: 1231–1235

van der Werf-Messing B (1973) Carcinoma of the kidney. *Cancer* **32**: 1056–1061

van der Wiel-van Kemenade E, Ligtenberg MJL, de Boer AJ, Buijs F, Vos HL, Melief CJM, Hilkens J and Figdor CG (1993) Episialin (MUC1) inhibits cytotoxic lymphocyte-target cell interaction. *J Immunol* **151**: 767–776

Wesseling J, van der Valk SW and Hilkens J (1996) A mechanism for inhibition of E-cadherin-mediated cell–cell adhesion by the membrane-associated mucin episialin/MUC1. *Mol Biol Cell* **7**: 565–577

Yamamoto M, Bhavanandan VP, Nakamori S and Irimura T (1996) A novel monoclonal antibody specific for sialylated MUC1 mucin. *Jpn J Cancer Res* **87**: 488–496

 *© Cancer Research Campaign 1999*
